# Supplementary material for: Diverse organ-specific localisation of a chemical defence, cyanogenic glycosides, in flowers of eleven species of Proteaceae
Source: PLoS One. 2023 Apr 27;18(4):e0285007. doi: 10.1371/journal.pone.0285007 (PMC10138830; doi:10.1371/journal.pone.0285007)
Supplement: S1 Table — Voucher accession number: MELU—The University of Melbourne Herbarium (Victoria, Australia), NE–NCW Beadle Herbarium, University of New England (New South Wales, Australia). (PDF) [file pone.0285007.s001.pdf]

Title: Diverse organ-specific localisation of a chemical defence, cyanogenic glycosides, in flowers of eleven species of Proteaceae

Authors: Edita Ritmejerytė[1,2,3*], Berin A Boughton[2,4], Michael J Bayly[2], Rebecca E Miller[1, 5*]

[1] School of Ecosystem and Forest Sciences, The University of Melbourne, Richmond, Victoria 3121, Australia

[2] School of BioSciences, The University of Melbourne, Parkville, Victoria 3010, Australia

[3] Australian Institute of Tropical Health and Medicine, James Cook University, Smithfield, Queensland 4878, Australia

[4] Australian National Phenome Centre, Murdoch University, Western Australia 6150, Australia

[5] Royal Botanic Gardens Victoria, South Yarra, Victoria 3141, Australia

* Corresponding authors: edita.ritmejeryte@jcu.edu.au (ER) and rebecca.miller@rbg.vic.gov.au (REM)

Running title: Interspecific variation in floral cyanogenesis in Proteaceae

S1 Table. Collection information and accession numbers for voucher specimens for the eleven Proteaceae species used in this study. Voucher accession number: MELU - The University of Melbourne Herbarium (Victoria, Australia), NE – NCW Beadle Herbarium, University of New England (New South Wales, Australia).

Species	Collector	Date collected	Herbarium voucher accession number
Buckinghamia celsissima F. Muell.	R. Miller, D. King	2/10/2016	MELUD113006a
Grevillea robusta A.Cunn. ex R.Br.	R. Miller	12/12/2017	MELUD117046a
Hakea bucculenta C. A. Gardner	R. Miller	6/27/2017	MELUD155100a
Helicia australasica F. Muell.	R. Miller	8/29/2017	MELUD117037a, MELUD117038a, MELUD117041a
Hollandea riparia B. Hyland	R. Miller	8/31/2017	MELUD113057a, MELUD113124a
Lasjia claudiensis C. L. Gross & B. Hyland	R. Miller	8/30/2017	MELUD117039a
Lomatia myricoides (C.F.Gaertn.) Domin	W. Grimm	31/01/2018	MELUD117049a
Macadamia tetraphylla L. A. S. Johnson	R. Miller	9/2/2017	MELUD117043a
Megahertzia amplexicaulis A. S. George & B. Hyland	B. Gray	8/11/2016	MELUD113058a
Neorites kevedianus L. S. Sm.	B. Gray	6/3/2016	MELUD113028a
Telopea speciosissima (Sm.) R. Br.	E. Ritmejerytė	11/1/2016	MELUD117842a; NE108552
