# Supplementary material for: Diverse organ-specific localisation of a chemical defence, cyanogenic glycosides, in flowers of eleven species of Proteaceae
Source: PLoS One. 2023 Apr 27;18(4):e0285007. doi: 10.1371/journal.pone.0285007 (PMC10138830; doi:10.1371/journal.pone.0285007)
Supplement: S2 Table — Letters (abc) indicate significant difference in floral or foliar log transformed content between species, using Tukey family grouping test, means that do not share a letter are significantly different. (PDF) [file pone.0285007.s002.pdf]

**Title:** Diverse organ-specific localisation of a chemical defence, cyanogenic glycosides, in flowers of eleven species of Proteaceae

**Authors:** Edita Ritmejerytė[1,2,3*], Berin A Boughton[2,4], Michael J Bayly[2], Rebecca E Miller[1, 5*]

[1] School of Ecosystem and Forest Sciences, The University of Melbourne, Richmond, Victoria 3121, Australia

[2] School of BioSciences, The University of Melbourne, Parkville, Victoria 3010, Australia

[3] Australian Institute of Tropical Health and Medicine, James Cook University, Smithfield, Queensland 4878, Australia

[4] Australian National Phenome Centre, Murdoch University, Western Australia 6150, Australia

[5] Royal Botanic Gardens Victoria, South Yarra, Victoria 3141, Australia

* Corresponding authors: edita.ritmejeryte@jcu.edu.au (ER) and rebecca.miller@rbg.vic.gov.au (REM)

**Running title:** Interspecific variation in floral cyanogenesis in Proteaceae

**S2 Table. The cyanogenic glycoside content of measured as evolved cyanide (µg CN $g^{-1}$ DW) from whole florets and leaves of eleven Proteaceae species (means ± SE, n = 3–5 replicate composite samples from 1-6 plants).** Letters (abc) indicate significant difference in floral or foliar log transformed content between species, using Tukey family grouping test, means that do not share a letter are significantly different.

| Species | Whole floret | | | | Leaf | | | |
|---|---|---|---|---|---|---|---|---|
| *Buckinghamia celsissima* | 2358.7 | ± | 139.6 | c | 0.2 | ± | 0.1 | g |
| *Grevillea robusta* | 5578.2 | ± | 78.8 | a | 6.2 | ± | 0.3 | e |
| *Hakea bucculenta* | 3359.3 | ± | 100.4 | bc | 1643.8 | ± | 112.6 | a |
| *Helicia australasica* | 3.6 | ± | 0.5 | h | 16.9 | ± | 0.3 | d |
| *Hollandaea riparia* | 23.1 | ± | 1.2 | g | 14.5 | ± | 1.6 | d |
| *Lasjia claudiensis* | 449.9 | ± | 39.6 | de | 13.9 | ± | 1.0 | d |
| *Lomatia myricoides* | 668.1 | ± | 103.9 | d | 1.8 | ± | 0.2 | f |
| *Macadamia tetraphylla* | 4280.6 | ± | 161.9 | ab | 12.5 | ± | 0.8 | de |
| *Megahertzia amplexicaulis* | 3936.7 | ± | 129.6 | ab | 84.0 | ± | 4.8 | c |
| *Neorites kevedianus* | 58.2 | ± | 4.3 | f | 2.4 | ± | 0.4 | f |
| *Telopea speciosissima* | 384.7 | ± | 29.9 | e | 204.0 | ± | 25.9 | b |
| ***F* (10, 26)** | **738.1** | | | | **362.7** | | | |
| ***P*** | **<0.0001** | | | | **<0.0001** | | | |
