# Supplementary material for: Diverse organ-specific localisation of a chemical defence, cyanogenic glycosides, in flowers of eleven species of Proteaceae
Source: PLoS One. 2023 Apr 27;18(4):e0285007. doi: 10.1371/journal.pone.0285007 (PMC10138830; doi:10.1371/journal.pone.0285007)
Supplement: S3 Table — Evolved cyanide content was significantly different in all species (P ≤ 0.001). Letters (abc) indicate significant difference between floral tissues for the same species, using Tukey family grouping test; means that do not share a letter are significantly different. ND–no data, i.e. for L. claudiensis and N. kevedianus the “pollen presenter” content is from style and pollen presenter combined, and for H. australasica, H. riparia, N. kevedianus, L. claudiensis and M. amplexicaulis anthers were analysed with perianth. NA–tissue not present for the species. (PDF) [file pone.0285007.s003.pdf]

Title: Diverse organ-specific localisation of a chemical defence, cyanogenic glycosides, in flowers of eleven species of Proteaceae

Authors: Edita Ritmejerytė[1,2,3*], Berin A Boughton[2,4], Michael J Bayly[2], Rebecca E Miller[1, 5*]

[1] School of Ecosystem and Forest Sciences, The University of Melbourne, Richmond, Victoria 3121, Australia

[2] School of BioSciences, The University of Melbourne, Parkville, Victoria 3010, Australia

[3] Australian Institute of Tropical Health and Medicine, James Cook University, Smithfield, Queensland 4878, Australia

[4] Australian National Phenome Centre, Murdoch University, Western Australia 6150, Australia

[5] Royal Botanic Gardens Victoria, South Yarra, Victoria 3141, Australia

* Corresponding authors: edita.ritmejeryte@jcu.edu.au (ER) and rebecca.miller@rbg.vic.gov.au (REM)

Running title: Interspecific variation in floral cyanogenesis in Proteaceae

S3 Table. The cyanogenic glycoside content of measured as evolved cyanide (μg CN g^{-1} DW) from floral tissues of eleven Proteaceae species (means ± SE, n = 3–5 replicate composite samples from 1-6 plants). Evolved cyanide content was significantly different in all species ($P \leq 0.001$). Letters (abc) indicate significant difference between floral tissues for the same species, using Tukey family grouping test; means that do not share a letter are significantly different. ND – no data, i.e. for *L. claudiensis* and *N. kevedianus* the "pollen presenter" content is from style and pollen presenter combined, and for *H. australasica*, *H. riparia*, *N. kevedianus*, *L. claudiensis* and *M. amplexicaulis* anthers were analysed with perianth. NA – tissue not present for the species.

Species	Floral tissue													
	Pedicel		Gynophore		Ovary		Style		Pollen presenter		Perianth		Anthers	
Buckinghamia celsissima	0.0 ± 0.0	d	NA		17.2 ± 0.9	c	0.0 ± 0.0	d	18.7 ± 2.0	c	1.5 ± 0.5	d	8088.6 ± 160.3	a
Grevillea robusta	4225.7 ± 117.6	c	11881.0 ± 370.7	a	9129.5 ± 89.1	a	9788.6 ± 201.9	a	5788.4 ± 136.3	b	3482.1 ± 34.4	c	171.8 ± 4.6	d
Hakea bucculenta	4550.4 ± 1009	ab	NA		5238.5 ± 363.2	a	5211.7 ± 208.1	a	1162.9 ± 224.3	b	3047.6 ± 116.4	ab	853.3 ± 151.2	b
Helicia australasica	1.3 ± 0.1	d	NA		3.6 ± 0.3	c	6.7 ± 0.9	bc	28.2 ± 2.0	a	1.9 ± 0.7	bcd	ND	
Hollandaea riparia	2.1 ± 0.4	b	NA		1.1 ± 0.1	b	1.4 ± 0.0	b	45.3 ± 12.3	a	27.7 ± 1.6	a	ND	
Lasjia claudiensis	268.3 ± 44.2	b	NA		1003.7 ± 156.9	a	ND		629.5 ± 16.8	ab	360.7 ± 46.0	b	ND	
Lomatia myricoides	38.0 ± 13.1	d	79.6 ± 15.2	d	331.3 ± 47.4	c	103.2 ± 6.0	d	2686.5 ± 107.4	b	85.2 ± 25.0	d	8000.6 ± 3017	a
Macadamia tetraphylla	2223.2 ± 590.8	a-e	NA		3096.6 ± 174.8	d	10048.3 ± 907.4	a	3159.4 ± 452.6	bcd	6430.7 ± 244.3	ab	1185.8 ± 111.2	e
Megahertzia amplexicaulis	1868.1 ± 58.0	d	NA		8937.3 ± 286.7	a	2923.3 ± 94.7	cd	2949.0 ± 286.1	bcd	3530.7 ± 184.2	bc	ND	
Neorites kevedianus	NA		NA		174.1 ± 13.4	a	ND		230.9 ± 46.4	a	23.4 ± 1.6	c	ND	
Telopea speciosissima	1019.7 ± 82.3	a	1050.5 ± 239.4	ab	372.3 ± 58.8	bc	6.0 ± 1.2	e	0.5 ± 0.1	f	1.2 ± 0.3	f	34.5 ± 5.7	d
