# Supplementary material for: Diverse organ-specific localisation of a chemical defence, cyanogenic glycosides, in flowers of eleven species of Proteaceae
Source: PLoS One. 2023 Apr 27;18(4):e0285007. doi: 10.1371/journal.pone.0285007 (PMC10138830; doi:10.1371/journal.pone.0285007)
Supplement: S4 Table — The dhurrin to proteacin ratios were derived from MALDI-MSI signal intensities. ND–no data (i.e. tissue was not available in the imaged section). NA–tissue not present/available for the species (i.e. spiral cells are only present in L. milnerae (syn L. fraxinifolia) and perianth/anthers not available in senescent floral stage). † L. milnerae data are from [17]; ‡ G. robusta and M. tetraphylla young floret data are from [18]. (PDF) [file pone.0285007.s004.pdf]

**Title:** Diverse organ-specific localisation of a chemical defence, cyanogenic glycosides, in flowers of eleven species of Proteaceae

**Authors:** Edita Ritmejerytė[1,2,3*], Berin A Boughton[2,4], Michael J Bayly[2], Rebecca E Miller[1, 5*]

[1] School of Ecosystem and Forest Sciences, The University of Melbourne, Richmond, Victoria 3121, Australia

[2] School of BioSciences, The University of Melbourne, Parkville, Victoria 3010, Australia

[3] Australian Institute of Tropical Health and Medicine, James Cook University, Smithfield, Queensland 4878, Australia

[4] Australian National Phenome Centre, Murdoch University, Western Australia 6150, Australia

[5] Royal Botanic Gardens Victoria, South Yarra, Victoria 3141, Australia

* Corresponding authors: edita.ritmejeryte@jcu.edu.au (ER) and rebecca.miller@rbg.vic.gov.au (REM)

**Running title:** Interspecific variation in floral cyanogenesis in Proteaceae

**S4 Table. Relative dhurrin and proteacin content of floral and fruit tissues from five Proteaceae taxa and across multiple developmental stages.** The dhurrin to proteacin ratios were derived from MALDI-MSI signal intensities. ND – no data (i.e. tissue was not available in the imaged section). NA – tissue not present/available for the species (i.e. spiral cells are only present in *L. milnerae* (syn *L. fraxinifolia*) and perianth/anthers not available in senescent floral stage). † *L. milnerae* data are from [1]; ‡ *G. robusta* and *M. tetraphylla* young floret data are from [2].

| Species and floral developmental stage | Dhurrin:proteacin ratio | | | | | | | | |
|---|---|---|---|---|---|---|---|---|---|
| | Floral tissue | | | | | | | | |
| | Pedicel | Gynophore | Ovary wall | Ovules | Style | Pollen presenter | Spiral cells | Anthers | Perianth |
| *L. milnerae* | | | | | | | | | |
| Partially open † | ND | 31 : 69 | 20 : 80 | 21 : 79 | 32 : 68 | 88 : 12 | 98 : 2 | 47 : 53 | 57 : 43 |
| *T. speciosissima* | | | | | | | | | |
| Open | 3 : 97 | 4 : 96 | 0 : 100 | 0 : 100 | 0 : 100 | 0 : 0 | NA | ND | ND |
| *M. tetraphylla* | | | | | | | | | |
| Young ‡ | ND | NA | ND | ND | 66 : 34 | 40 : 60 | NA | 18 : 82 | 57 : 43 |
| Partially open | 67 : 33 | NA | 26 : 74 | ND | 47 : 53 | ND | NA | ND | 49 : 51 |
| Open | 67 : 33 | NA | ND | ND | 52 : 48 | ND | NA | ND | 55 : 45 |
| *G. robusta* | | | | | | | | | |
| Young ‡ | 82 : 18 | NA | ND | ND | 76 : 24 | 74 : 26 | NA | 60 : 40 | 71 : 29 |
| Partially open | ND | 89 : 11 | 74 : 26 | 69 : 31 | ND | ND | NA | ND | 73 : 27 |
| Senescent | ND | 83 : 17 | 80 : 20 | 34 : 66 | 59 : 41 | 58 : 42 | NA | NA | NA |
| | Fruit tissue | | | | | | | | |
| | Pedicel | Pericarp | Locular cavity | Embryo | Style | Pollen presenter | Seed wing | | |
| *G. robusta* | | | | | | | | | |
| Fruit | 31 : 69 | 31 : 69 | 87 : 13 | 72 : 28 | ND | ND | 97 : 3 | | |
| *N. kevedianus* | | | | | | | | | |
| Fruit | NA | 9 : 91 | 7 : 93 | 4 : 96 | 14 : 86 | 4 : 96 | ND | | |

**References**

1. Ritmejerytė E, Boughton BA, Bayly MJ, Miller RE. Unique and highly specific cyanogenic glycoside localisation in stigmatic cells and pollen in the genus *Lomatia* (Proteaceae). Annals of Botany. 2020;126(3):387-400. doi: 10.1093/aob/mcaa038.

2. Ritmejerytė E, Miller RE, Bayly MJ, Boughton BA. Visualisation of cyanogenic glycosides in floral tissues. In: Beale DJ, Hillyer K, Warden AC, Jones OAH, editors. Applied Environmental Metabolomics: Community Insights and Guidance from the Field. 1 ed: Academic Press; 2022.
