# Supplementary material for: Diverse organ-specific localisation of a chemical defence, cyanogenic glycosides, in flowers of eleven species of Proteaceae
Source: PLoS One. 2023 Apr 27;18(4):e0285007. doi: 10.1371/journal.pone.0285007 (PMC10138830; doi:10.1371/journal.pone.0285007)
Supplement: S2 Fig — Each point indicates a biological replicate mean of 4 replicates sampled from each individual for each tissue. The mean whole floret content across the six individuals was 3530.18 ± 252.3 μg CN g-1 DW. Evolved cyanide content (or relative content) differed significantly between floral tissues (P ≤ 0.0001). Letters (abc) indicate significant differences between floral tissues, using Tukey HSD family grouping test; means that do not share a letter are significantly different. (PDF) [file pone.0285007.s006.pdf]

Title: Diverse organ-specific localisation of a chemical defence, cyanogenic glycosides, in flowers of eleven species of Proteaceae

Authors: Edita Ritmejerytė[1,2,3*], Berin A Boughton[2,4], Michael J Bayly[2], Rebecca E Miller[1, 5*]

[1] School of Ecosystem and Forest Sciences, The University of Melbourne, Richmond, Victoria 3121, Australia

[2] School of BioSciences, The University of Melbourne, Parkville, Victoria 3010, Australia

[3] Australian Institute of Tropical Health and Medicine, James Cook University, Smithfield, Queensland 4878, Australia

[4] Australian National Phenome Centre, Murdoch University, Western Australia 6150, Australia

[5] Royal Botanic Gardens Victoria, South Yarra, Victoria 3141, Australia

* Corresponding authors: edita.ritmejeryte@jcu.edu.au (ER) and rebecca.miller@rbg.vic.gov.au (REM)

Running title: Interspecific variation in floral cyanogenesis in Proteaceae

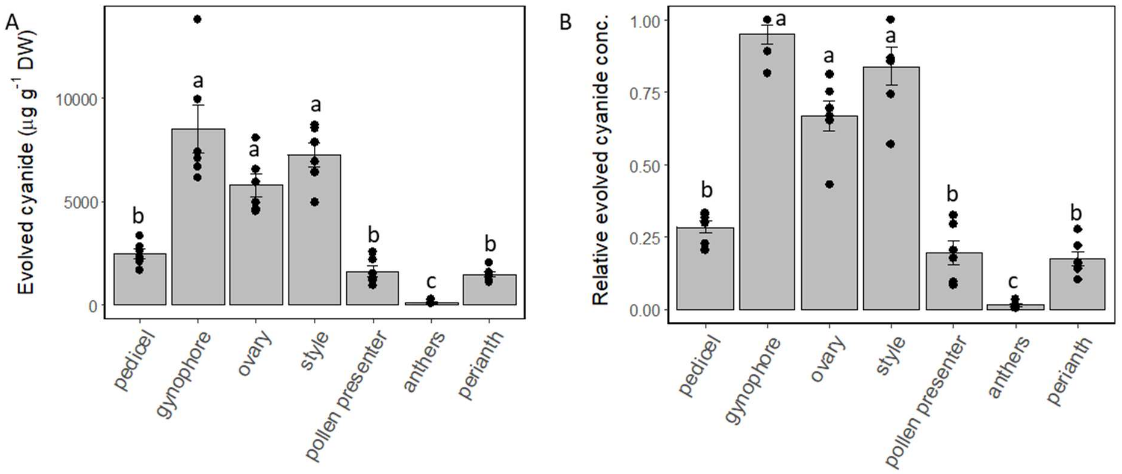

S2 Fig. (A) The variation of tissue-specific floral evolved cyanide content (µg CN g^{-1} DW) and (B) relative evolved cyanide content of six *Grevillea robusta* biological replicates (means ± SE) at partially open stage. Each point indicates a biological replicate mean of 4 replicates sampled from each individual for each tissue. The mean whole floret content across the six individuals was 3530.18 ± 252.3 µg CN g^{-1} DW. Evolved cyanide content (or relative content) differed significantly between floral tissues ($P \leq 0.0001$). Letters (abc) indicate significant differences between floral tissues, using Tukey HSD family grouping test; means that do not share a letter are significantly different.
