# Supplementary material for: Diverse organ-specific localisation of a chemical defence, cyanogenic glycosides, in flowers of eleven species of Proteaceae
Source: PLoS One. 2023 Apr 27;18(4):e0285007. doi: 10.1371/journal.pone.0285007 (PMC10138830; doi:10.1371/journal.pone.0285007)
Supplement: S3 Fig — Mass errors (Δm) were typically ≤ 2 mDa. (PDF) [file pone.0285007.s007.pdf]

Title: Diverse organ-specific localisation of a chemical defence, cyanogenic glycosides, in flowers of eleven species of Proteaceae

Authors: Edita Ritmejerytė[1,2,3*], Berin A Boughton[2,4], Michael J Bayly[2], Rebecca E Miller[1, 5*]

[1] School of Ecosystem and Forest Sciences, The University of Melbourne, Richmond, Victoria 3121, Australia

[2] School of BioSciences, The University of Melbourne, Parkville, Victoria 3010, Australia

[3] Australian Institute of Tropical Health and Medicine, James Cook University, Smithfield, Queensland 4878, Australia

[4] Australian National Phenome Centre, Murdoch University, Western Australia 6150, Australia

[5] Royal Botanic Gardens Victoria, South Yarra, Victoria 3141, Australia

* Corresponding authors: edita.ritmejeryte@jcu.edu.au (ER) and rebecca.miller@rbg.vic.gov.au (REM)

Running title: Interspecific variation in floral cyanogenesis in Proteaceae

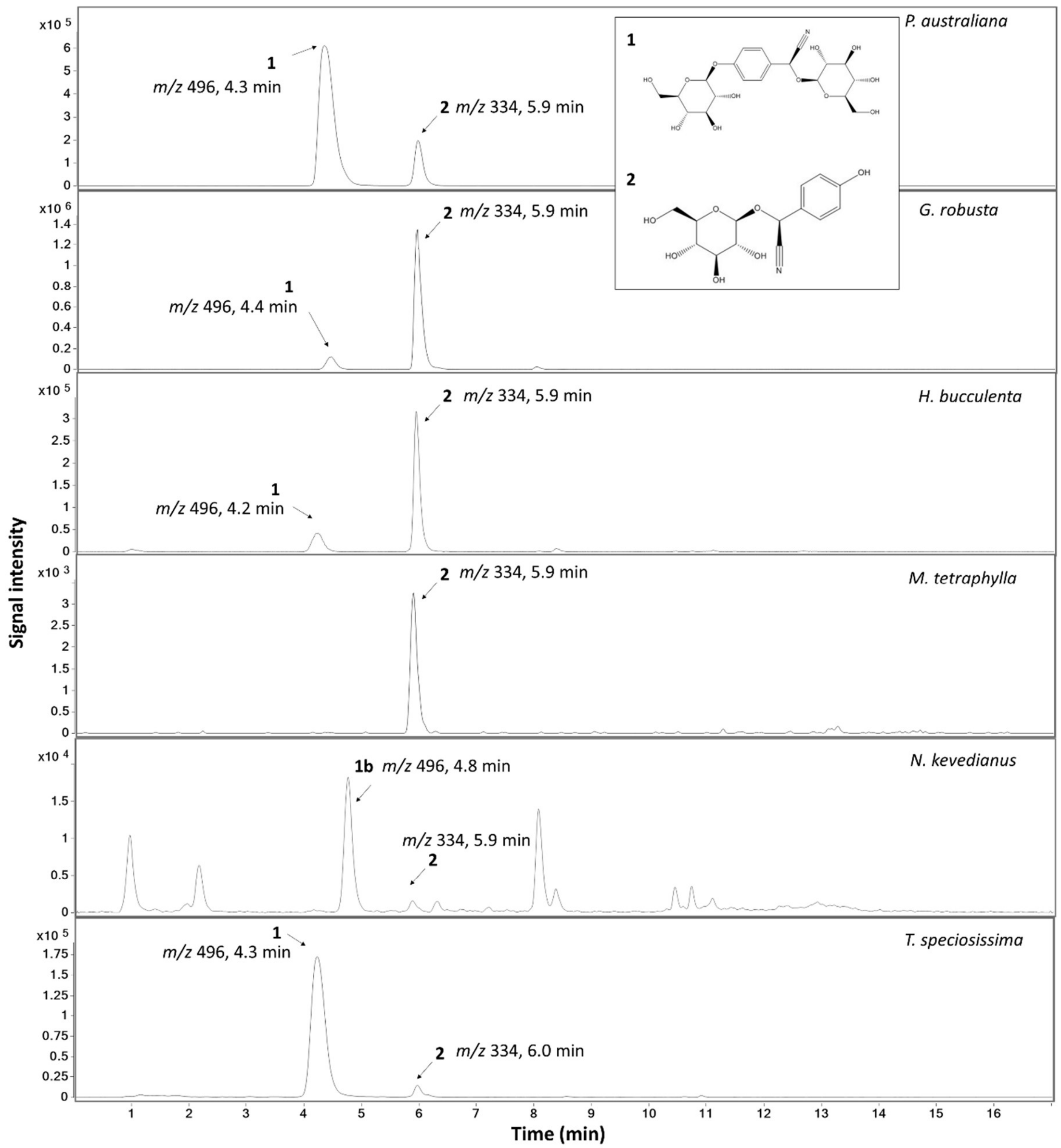

S3 Fig. Representative extracted ion chromatograms (EIC) from LC-MS corresponding to [M + Na]$^+$ proteacin (1) at *m/z* 496, and dhurrin (2) at *m/z* 334 in foliar extracts of *P. australiana* and *M. tetraphylla* and whole flower extracts of *G. robusta, H. bucculenta, N. kevedianus* and *T. speciosissima*. Mass errors (Δm) were typically ≤ 2 mDa.
