# Supplementary material for: Diverse organ-specific localisation of a chemical defence, cyanogenic glycosides, in flowers of eleven species of Proteaceae
Source: PLoS One. 2023 Apr 27;18(4):e0285007. doi: 10.1371/journal.pone.0285007 (PMC10138830; doi:10.1371/journal.pone.0285007)
Supplement: S4 Fig — Bars are means ± SE. Letters (abc) indicate significant differences at P < 0.05 between floral tissues, using Tukey-Kramer post-hoc test; means that do not share a letter are significantly different. NA–tissue not available for the group. (PDF) [file pone.0285007.s008.pdf]

**Title:** Diverse organ-specific localisation of a chemical defence, cyanogenic glycosides, in flowers of eleven species of Proteaceae

**Authors:** Edita Ritmejerytė[1,2,3*], Berin A Boughton[2,4], Michael J Bayly[2], Rebecca E Miller[1, 5*]

[1] School of Ecosystem and Forest Sciences, The University of Melbourne, Richmond, Victoria 3121, Australia

[2] School of BioSciences, The University of Melbourne, Parkville, Victoria 3010, Australia

[3] Australian Institute of Tropical Health and Medicine, James Cook University, Smithfield, Queensland 4878, Australia

[4] Australian National Phenome Centre, Murdoch University, Western Australia 6150, Australia

[5] Royal Botanic Gardens Victoria, South Yarra, Victoria 3141, Australia

* Corresponding authors: edita.ritmejeryte@jcu.edu.au (ER) and rebecca.miller@rbg.vic.gov.au (REM)

**Running title:** Interspecific variation in floral cyanogenesis in Proteaceae

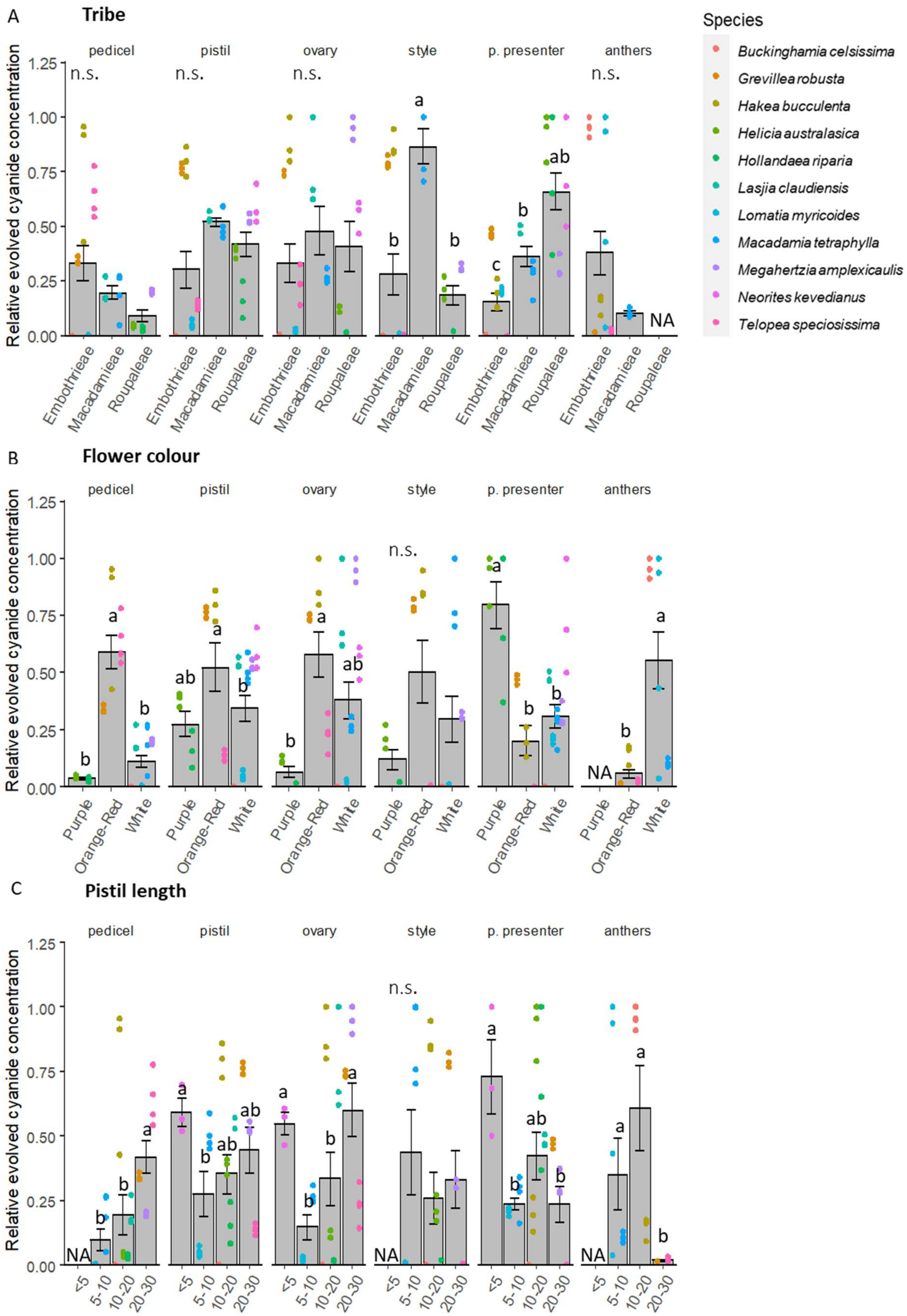

A
Tribe
B
Flower colour
C
Pistil length
pedicel
pistil
ovary
style
p. presenter
anthers
Relative evolved cyanide concentration
1.25
1.00
0.75
0.50
0.25
0.00
n.s.
a
b
ab
c
NA
Embothrieae
Macadamieae
Roupaleae
Purple
Orange-Red
White
<5
5-10
10-20
20-30
Species
Buckinghamia celsissima
Grevillea robusta
Hakea bucculenta
Helicia australasica
Hollandaea riparia
Lasjia claudiensis
Lomatia myricoides
Macadamia tetraphylla
Megahertzia amplexicaulis
Neorites kevedianus
Telopea speciosissima

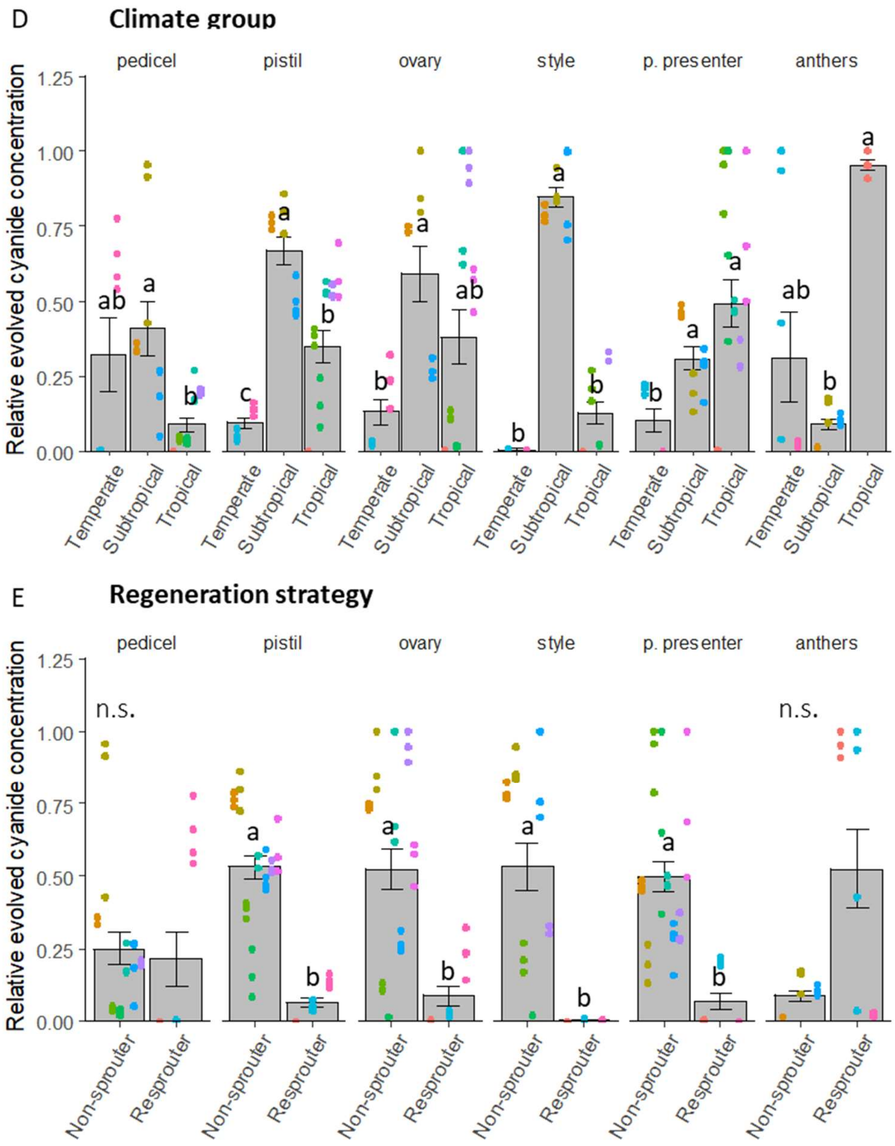


**S4 Fig. The relative content of evolved cyanide in specific floral tissues from 11 Proteaceae species grouped by (A) tribe, (B) flower colour, (C) pistil length (mm), (D) climate group, and (E) regeneration strategy.** Bars are means ± SE. Letters (abc) indicate significant differences at $P < 0.05$ between floral tissues, using Tukey-Kramer *post-hoc* test; means that do not share a letter are significantly different. NA – tissue not available for the group.
