# Supplementary material for: Diverse organ-specific localisation of a chemical defence, cyanogenic glycosides, in flowers of eleven species of Proteaceae
Source: PLoS One. 2023 Apr 27;18(4):e0285007. doi: 10.1371/journal.pone.0285007 (PMC10138830; doi:10.1371/journal.pone.0285007)
Supplement: Attachment — Submitted filename: Ritmejeryte et al 2023_Response to Reviewers.docx [file pone.0285007.s010.docx]

Re: Manuscript ID PONE-D-22-02128R2

Interspecific differences in floral chemical defence: diverse organ-specific localisation of cyanogenic glycosides in flowers of Proteaceae

PLOS ONE

Dear Dr. Ritmejerytė,

Thank you for submitting your manuscript to PLOS ONE. After careful consideration, we feel that it has merit but does not fully meet PLOS ONE’s publication criteria as it currently stands. Therefore, we invite you to submit a revised version of the manuscript that addresses the points raised during the review process.

==============================

Although reviewers' comments are carefully addresses, several minor editorial corrections should be made before proceeding.

The first is the use of the term "concentration(s)". Since cyanogenic glycosides are expressed as μg per g tissue DW but not in a certain volume, "concentration(s)" should be substituted by "content". Please see e.g., 10.11613/BM.2013.017 for further information.

**Authors response:** we thank the editor for noting this, however we believe that the term “concentration” is more appropriate in this specific instance for the following reasons. Firstly, vast majority of literature on cyanogenesis (and other plant defences) use the term “concentrations” for w/w measures, including the original methodology papers on quantifying the cyanogenic glycosides in plant tissues (µg CN per g tissue DW) (Brinker and Seigler 1989, Gleadow et al. 2011), as well as more recent papers in the field of cyanogenesis (e.g. Myrans et al. 2021, Pinheiro De Castro et al. 2019), and other plant defences (e.g. Goodger et al. 2016, Martinez-Swatson et al. 2020). Concentration is the most relevant measure for quantifying plant toxicity and relating it to the dosis letalis specific to a particular herbivore (Nahrstedt, 1985), whereas the term “content” is used to indicate the absolute amount of cyanide (or cyanogenic glycosides) in the entire plant or a particular plant organ (Jørgensen et al. 2005).

References:

Brinker, A. M. and D. S. Seigler (1989). "Methods for the detection and quantitative determination of cyanide in plant materials." Phytochemical Bulletin **21**(2): 24-31.

Gleadow, R. M., et al. (2011). Cyanogenic glycosides. Research methods in plant sciences. S. S. Narwal, L. Szajdak and D. A. Sampietro. USA, Stadium Press LLC. **1:** 283-310.

Myrans, H., et al. (2021). "Nitrogen availability and allocation in sorghum and its wild relatives: divergent roles for cyanogenic glucosides." Journal of Plant Physiology: 153393.

Pinheiro De Castro, É. C., et al. (2019). "Sequestration and biosynthesis of cyanogenic glucosides in passion vine butterflies and consequences for the diversification of their host plants." Ecology and Evolution **9**(9): 5079-5093.

Goodger, J. Q. D., et al. (2016). "Foliar essential oil glands of Eucalyptus subgenus Eucalyptus (Myrtaceae) are a rich source of flavonoids and related non-volatile constituents." PLoS ONE **11**(3).

Martinez-Swatson, K., et al. (2020). "Exploring evolutionary theories of plant defence investment using field populations of the deadly carrot." Annals of Botany **125**(5): 737-750.

Nahrstedt, A. (1985). "Cyanogenic compounds as protecting agents for organisms." Plant Systematics and Evolution **150**(1-2): 35-47.

Jørgensen, K., et al. (2005). "Cassava plants with a depleted cyanogenic glucoside content in leaves and tubers. Distribution of cyanogenic glucosides, their site of synthesis and transport, and blockage of the biosynthesis by RNA interference technology." Plant Physiology **139**: 363-374.

Please avoid using vernacular phrases such as e.g., "florets of 11 Proteaceae" or "in flowers of Proteaceae" but use scientifically acceptable terminology and do not spare words to describe an object of the study, for example "florets of 11 studied species from (belonging to) the Proteaceae family" or "in flowers of (studied) species from the Proteaceae family".

**Authors response:** we thank the editor for noting this. We have changed the wording throughout the manuscript to reflect this comment which can be seen as tracked changes.

The main title might be misleading and a reader might conclude the authors report a review article of most recent literature regarding the content of cyanogenic glycosides across more than 80 genera of the Proteaceae family. Please specify here how many species you studied, for example: "...in flowers of 11 species from the family Proteaceae".

**Authors response:** we thank the editor for pointing this out. The number of species has been added to the main title and now reads “Diverse organ-specific localisation of a chemical defence, cyanogenic glycosides, in flowers of eleven species of Proteaceae”.

Please also consider a minor remark by Reviewer #2 related to L300.

**Authors response:** we thank the reviewer for noting this, the text has been corrected to “general linear model”.

==============================

Please submit your revised manuscript by May 11 2023 11:59PM. If you will need more time than this to complete your revisions, please reply to this message or contact the journal office at [plosone@plos.org](mailto:plosone@plos.org). When you're ready to submit your revision, log on to https://www.editorialmanager.com/pone/ and select the 'Submissions Needing Revision' folder to locate your manuscript file.

Please include the following items when submitting your revised manuscript:

- A rebuttal letter that responds to each point raised by the academic editor and reviewer(s). You should upload this letter as a separate file labeled 'Response to Reviewers'.
- A marked-up copy of your manuscript that highlights changes made to the original version. You should upload this as a separate file labeled 'Revised Manuscript with Track Changes'.
- An unmarked version of your revised paper without tracked changes. You should upload this as a separate file labeled 'Manuscript'.

If you would like to make changes to your financial disclosure, please include your updated statement in your cover letter. Guidelines for resubmitting your figure files are available below the reviewer comments at the end of this letter.

If applicable, we recommend that you deposit your laboratory protocols in protocols.io to enhance the reproducibility of your results. Protocols.io assigns your protocol its own identifier (DOI) so that it can be cited independently in the future. For instructions see: <https://journals.plos.org/plosone/s/submission-guidelines#loc-laboratory-protocols>. Additionally, PLOS ONE offers an option for publishing peer-reviewed Lab Protocol articles, which describe protocols hosted on protocols.io. Read more information on sharing protocols at <https://plos.org/protocols?utm_medium=editorial-email&utm_source=authorletters&utm_campaign=protocols>.

We look forward to receiving your revised manuscript.

Kind regards,

Branislav T. Šiler, Ph.D.

Academic Editor

PLOS ONE

Journal Requirements:

Please review your reference list to ensure that it is complete and correct. If you have cited papers that have been retracted, please include the rationale for doing so in the manuscript text, or remove these references and replace them with relevant current references. Any changes to the reference list should be mentioned in the rebuttal letter that accompanies your revised manuscript. If you need to cite a retracted article, indicate the article’s retracted status in the References list and also include a citation and full reference for the retraction notice.

[Note: HTML markup is below. Please do not edit.]

Reviewers' comments:

Reviewer's Responses to Questions

**Comments to the Author**

1. If the authors have adequately addressed your comments raised in a previous round of review and you feel that this manuscript is now acceptable for publication, you may indicate that here to bypass the “Comments to the Author” section, enter your conflict of interest statement in the “Confidential to Editor” section, and submit your "Accept" recommendation.

Reviewer #1: All comments have been addressed

Reviewer #2: All comments have been addressed

2. Is the manuscript technically sound, and do the data support the conclusions?

The manuscript must describe a technically sound piece of scientific research with data that supports the conclusions. Experiments must have been conducted rigorously, with appropriate controls, replication, and sample sizes. The conclusions must be drawn appropriately based on the data presented.

Reviewer #1: Yes

Reviewer #2: Yes

3. Has the statistical analysis been performed appropriately and rigorously?

Reviewer #1: Yes

Reviewer #2: Yes

4. Have the authors made all data underlying the findings in their manuscript fully available?

The [PLOS Data policy](http://www.plosone.org/static/policies.action#sharing) requires authors to make all data underlying the findings described in their manuscript fully available without restriction, with rare exception (please refer to the Data Availability Statement in the manuscript PDF file). The data should be provided as part of the manuscript or its supporting information, or deposited to a public repository. For example, in addition to summary statistics, the data points behind means, medians and variance measures should be available. If there are restrictions on publicly sharing data—e.g. participant privacy or use of data from a third party—those must be specified.

Reviewer #1: Yes

Reviewer #2: Yes

5. Is the manuscript presented in an intelligible fashion and written in standard English?

PLOS ONE does not copyedit accepted manuscripts, so the language in submitted articles must be clear, correct, and unambiguous. Any typographical or grammatical errors should be corrected at revision, so please note any specific errors here.

Reviewer #1: Yes

Reviewer #2: Yes

6. Review Comments to the Author

Please use the space provided to explain your answers to the questions above. You may also include additional comments for the author, including concerns about dual publication, research ethics, or publication ethics. (Please upload your review as an attachment if it exceeds 20,000 characters)

Reviewer #1: (No Response)

Reviewer #2: One minor thing: ref Line 300: Was this a general linear model, or generalized linear model? I think general linear mixed model?

**Authors response:** we thank the reviewer for noting this, the text has been corrected to “general linear model”.

7. PLOS authors have the option to publish the peer review history of their article ([what does this mean?](https://journals.plos.org/plosone/s/editorial-and-peer-review-process#loc-peer-review-history)). If published, this will include your full peer review and any attached files.


If you choose “no”, your identity will remain anonymous but your review may still be made public.


**Do you want your identity to be public for this peer review?** For information about this choice, including consent withdrawal, please see our [Privacy Policy](https://www.plos.org/privacy-policy).

Reviewer #1: No

Reviewer #2: No

[NOTE: If reviewer comments were submitted as an attachment file, they will be attached to this email and accessible via the submission site. Please log into your account, locate the manuscript record, and check for the action link "View Attachments". If this link does not appear, there are no attachment files.]

While revising your submission, please upload your figure files to the Preflight Analysis and Conversion Engine (PACE) digital diagnostic tool, <https://pacev2.apexcovantage.com/>. PACE helps ensure that figures meet PLOS requirements. To use PACE, you must first register as a user. Registration is free. Then, login and navigate to the UPLOAD tab, where you will find detailed instructions on how to use the tool. If you encounter any issues or have any questions when using PACE, please email PLOS at [figures@plos.org](mailto:figures@plos.org). Please note that Supporting Information files do not need this step.

*In compliance with data protection regulations, you may request that we remove your personal registration details at any time.* [*(Remove my information/details)*](https://www.editorialmanager.com/pone/login.asp?a=r)*. Please contact the publication office if you have any questions.*
